# Discrimination Factor of Sulphur Stable Isotope Ratios Between Pregnant Fin Whales and Their Foetuses

**DOI:** 10.1002/rcm.10057

**Published:** 2025-04-28

**Authors:** Marc Ruiz‐Sagalés, Asunción Borrell, Alex Aguilar

**Affiliations:** ^1^ Institut de Recerca de la Biodiversitat (IRBio) and Departament de Biologia Evolutiva, Ecologia i Ciències Ambientals (BEECA), Facultat de Biologia Universitat de Barcelona Barcelona Spain; ^2^ Reial Acadèmia de Ciències i Arts de Barcelona (RACAB) Barcelona Spain

**Keywords:** foetal development, foetus–mother discrimination, gestation, muscle, North Atlantic, *δ* ^34^S values

## Abstract

**Rationale:**

In‐utero synthesised tissues of mammals have often been used to infer maternal behaviour during gestation. Differences in *δ* ^15^N or *δ* ^13^C values between foetal and maternal tissues (foetus–mother discrimination factors) are well established, but they remain uncertain for *δ* ^34^S values. This study addresses this gap by investigating such discrimination in *δ* ^34^S values of fin whale muscle ( *Balaenoptera physalus* ) and its potential variation throughout gestation.

**Methods:**

We analysed muscle *δ* ^34^S values in 11 pregnant fin whales and their respective foetuses. Samples were obtained from individuals feeding off northwestern (NW) Spain during the 1983–1985 summer seasons. Yearday (0–365) and foetal length at the moment of sampling were considered proxies of the gestation stage and their effect on discrimination factors was examined. The *δ* ^34^S values were determined by continuous flow isotope ratio mass spectrometry.

**Results:**

*δ* ^34^S values in foetal and maternal muscle were positively correlated. *δ* ^34^S values were higher in foetal muscle (M ± SD = 19.2 ± 0.3 ‰) compared to the maternal one (M ± SD = 18.6 ± 0.4 ‰), with a foetus–mother discrimination of Δ^34^S = 0.59 ± 0.15 ‰. This observed enrichment may be due to differences in isotopic turnover rates, amino acid metabolism, and/or maternal dietary patterns during gestation. The foetus–mother *δ* ^34^S discrimination values did not change with yearday or foetus length.

**Conclusions:**

These findings are relevant for understanding foetal‐maternal *δ* ^34^S discrimination and drawing ecological inferences from foetal tissues. Further research is needed to understand the mechanisms driving *δ* ^34^S fractionation under different scenarios.

## 1 Introduction

While nitrogen and carbon stable isotope ratios (*δ* ^15^N and *δ* ^13^C) have been widely applied to ecological studies in marine mammals [1], the use of sulphur stable isotope ratios (*δ* ^34^S) remains less common despite providing complementary information to that obtained from those of nitrogen and carbon. This lesser use makes the physiological pathways and the inter‐individual and intra‐individual variability of this ratio poorly known.

Sulphur isotopes can produce insights into physiological processes, such as the determination of the maternal influence on the isotopic composition of slow turnover tissues (i.e., muscle) in juvenile sharks [2]. Early tissues reflect maternal contributions from gestation or incubation, and as juveniles grow and forage independently, different organs retain maternal signatures according to their turnover rates. Also, sulphur isotopes contribute to identifying dietary sources by improving the separation of multiple food sources within complex food webs [3]. They also enhance the understanding of trophic interactions by allowing finer niche differentiation when used in conjunction with nitrogen and/or carbon stable isotope ratios [4], clarify habitat use through the distinction between benthic and pelagic contributions [5] or reveal habitat use patterns across terrestrial–marine gradients [6]. Sulphur stable isotope ratios also exhibit relatively low trophic discrimination factors (TDFs), that is, small differences in *δ* ^34^S values between food sources and consumers as compared to environmental baselines, although these TDFs can vary across taxa [7]. Variations may relate to dietary protein content and dietary *δ* ^34^S values, with higher protein diets increasing *δ* ^34^S discrimination and dietary *δ* ^34^S values being positively related to consumer *δ* ^34^S values, as well as inversely related to *δ* ^34^S discrimination [8, 9].

In mammals, TDFs for sulphur stable isotope ratios are generally low (M ± SD = −0.6 ± 1.3‰) [3], a fact that allows ecological tracing with limited trophic influence. Unlike nitrogen stable isotope ratios, where the metabolism of multiple amino acids contributes to the TDF, sulphur stable isotope ratios depend upon the metabolism of the only two amino acids that contain this element: methionine and cysteine. Methionine is an essential amino acid and thus derives only from the diet [10] except in ruminants, in which rumen microbes can synthesise it [11]. Thus, the sulphur stable isotope ratio of methionine in the body tissues should mirror that of the ingested methionine. Conversely, cysteine is a semi‐essential amino acid, meaning that it can be incorporated into body tissues both through the diet and through the metabolism of methionine or serine [12]. This mixed origin also determines a mixed sulphur stable isotope composition.

In humans, *δ* ^15^N and *δ* ^13^C values in hairs from newborns (in‐utero synthesised) and in those from their mothers are positively correlated [13]. This has prompted the use of tissues deposited during the foetal period to identify maternal food sources and infer habitat use during gestation in other mammals [14]. The difference in isotopic values between foetuses and their mothers is known as the foetus–mother discrimination (F‐MD) factor. It remains unclear whether it changes throughout gestation. In southern elephant seals ( *Mirounga leonina* ), *δ* ^15^N and *δ* ^13^C values in whiskers of offspring (in‐utero synthesised) and in those of their mothers exhibited both positive and negative correlations during gestation [15]. This variation among individuals may result from differences in foraging success and associated maternal body condition [15]. In the same study, *δ* ^15^N values of trophic and source amino acids were similar in the whiskers of offspring (in‐utero synthesised) and mothers during the first and second trimester of gestation compared to the third trimester [15]. This highlights that the effect of gestation on the isotopic values of in‐utero synthesised tissues and of mother tissues is not yet well understood. F‐MD in *δ* ^15^N and *δ* ^13^C values have been investigated across several marine mammals. For instance, muscle from fin whale ( *Balaenoptera physalus* ) foetuses were enriched in ^15^N (Δ^15^N = 1.5 ‰) and ^13^C (Δ^13^C = 1.1 ‰) compared to their mothers [16]. Contrary to the diversity of species and studies carried out on these two elements, a F‐MD factor for *δ* ^34^S values has not been estimated yet, to our knowledge, for any marine mammal species.

The life cycle of the fin whale is broadly representative of the typical patterns observed in most mysticetes. Thus, most fin whale populations are known to carry out migratory movements during which they aggregate during the summer in high‐latitude feeding grounds, where they primarily consume euphausiids, and migrate to low‐latitude breeding grounds in the winter. During the latter period, fin whales disperse and significantly reduce their feeding activity [17]. Because of this marked variation in the seasonal intensity of prey uptake, they are sometimes classified as capital breeders (e.g., [18]). Fin whales also exhibit a 2‐year reproduction cycle that is characterised by copulation in winter and giving birth during the following winter, both events occurring in the breeding grounds [19].

Considering the representativeness of the fin whale's life cycle in relation to the general behaviour of balaenopterids, we selected this species as a model that can potentially be extended to other species with similar habits. Taking advantage of a unique set of maternal and foetal tissue samples collected in the 1980s and preserved frozen in a tissue bank, we investigated foetal‐maternal *δ* ^34^S F‐MD in muscle in this species and assessed its variation throughout foetal growth.

## 2 Methods

### 2.1 Sample Collection

Muscle samples were collected from 11 pregnant fin whales, referred to hereafter as mothers, as well as from their corresponding foetuses. The sampled individuals had been caught off northwestern (NW) Spain during the 1983–1985 whaling seasons and flensed at the Caneliñas whaling station (Galicia, Spain).

In fin whales, the body length of the foetuses typically adjusts to a nearly parabolic curve with gestation month [20]. Body length measurements of mothers and foetuses were recorded at the time of sample collection, and yearday was calculated based on the day of the year the whales were caught (ranging from 1 for 1 January to 365 for 31 December). The sampling does not cover the entire gestation period, which lasts about 11 months but is representative of the gestational stage during the covered period (about 3 months). After collection, the muscle samples were preserved at −20°C in the BMA Tissue Bank of the University of Barcelona until analysis.

After thawing, foetal and maternal muscle samples were dried at 60°C for 48 h. To prevent lipids from altering other isotopic analytical results, the lipophilic fraction was removed using a chloroform‐methanol (2:1) solution following the Folch method [21]. However, we expected that lipid extraction did not affect *δ* ^34^S values, as it has been seen to be the case in similar studies conducted in the skin of other marine mammals [22]. Samples were dried again at 60°C for 24 h and ground into powder using a mortar and a pestle.

### 2.2 Stable Isotope Analyses

We weighed 2.5 ± 0.5 mg of each muscle sample and placed it in tin capsules for combustion at 1030°C. Analyses were conducted using a continuous‐flow isotope‐ratio mass spectrometry system comprising an Elemental Analyser (Carlo Erba 1108) coupled to a Delta Plus XP isotope ratio mass spectrometer (Thermo Fisher Scientific, Bremen, Germany). All analyses were performed at the Centres Cientifics i Tecnològics of the University of Barcelona (CCiT‐UB; Barcelona, Spain).

Analytical results are expressed using the *δ* notation, where the variation of stable isotope ratios is expressed in per mil (‰) relative to predefined standards:

$$\delta X\left(‰\right)=\left(\frac{R_{\text{sample}}}{R_{\text{standard}}}-1\right)\times 1000$$

Here, X represents ^34^S and R_sample_ and R_standard_ are the heavy‐to‐light stable isotope ratios (^34^S/^32^S) in the sample and the reference standards. The reference standard used is Vienna Canyon Diablo Troilite (V‐CDT). International isotope secondary standards of known ^34^S/^32^S ratios relative to V‐CDT were used to calibrate the system and compensate for any analytical drift over time. These standards include barium sulphate (NBS127; *δ* ^34^S = +20.3 ‰ and IAEA‐SO‐6; *δ* ^34^S = −34.1 ‰), barium sulphate salt (IAEA‐SO‐5; *δ* ^34^S = +0.5 ‰) and YCEM (*δ* ^34^S = +12.8 ‰). The analytical precision of the measurements was 0.2 ‰. The wt % S ranged between 0.6 and 0.9, values that are above the consensus threshold of > 0.1 wt % S required to conduct a reliable analysis.

### 2.3 Data Analysis

*δ* ^34^S values were tested for normality using Lilliefors's test and for homoscedasticity using Levene's test. We applied a first linear model (LM) to evaluate the correlation in *δ* ^34^S values between foetuses and mothers and conducted a pairwise t‐test with independent variance estimates for each group to assess differences in *δ* ^34^S values. Moreover, to assess the influence of each group on *δ* ^34^S values while controlling for within foetus–mother pair variability, we also applied a linear mixed‐effects model (LMM). This model included class (foetus or mother) as the fixed effect and paired foetus–mother groups as the random effect. To estimate the F‐MD for *δ* ^34^S values, we calculated the mean difference in muscle *δ* ^34^S values between foetuses and their mothers. We also computed the combined standard deviation to quantify the variability of this difference. Finally, to examine the F‐MD variation throughout gestation, we fitted other two LMs with the F‐MD of each foetus–mother pair as the response variable, one with the yearday (0–365) and the other with the foetus length as the predictors. All analyses were performed using R (version 4.4.1) [23].

## 3 Results

The foetuses generally exhibited higher *δ* ^34^S values (M = 19.2 ‰; SD = 0.3 ‰) compared to their mothers (M = 18.6 ‰; SD = 0.4 ‰; Table 1), as revealed by the pairwise *t*‐test (*t* = 3.86, *df* = 19.7, *p* = 0.001). The LMM further supported these differences in *δ* ^34^S values (*R* ^2^m = 0.42; *R* ^2^c = 0.85, *p* = 1.62 × 10^−5^).

**TABLE 1:** Information and muscle *δ* ^34^S values of the studied fin whale foetus–mother pairs.

Number	Date	Mother length (m)	Foetus sex	Foetus length (m)	Mother *δ* ^34^S values (‰)	Foetus *δ* ^34^S values (‰)	F‐MD *δ* ^34^S values (‰)	Mother %S	Foetus %S
83 127	27/08/83	20.80	Female	4.40	18.4	19.2	0.8	0.6	0.7
83 134	07/09/83	20.40	Male	2.70	18.4	19.0	0.6	0.7	0.7
83 158	23/09/83	18.20	Female	2.63	18.7	19.4	0.7	0.7	0.7
84 006	04/07/84	20.70	Male	2.80	18.1	18.7	0.6	0.7	0.7
84 033	23/07/84	17.60	Female	1.65	18.5	19.5	1.0	0.7	0.9
84 038	29/07/84	19.40	Female	1.61	18.9	19.6	0.7	0.8	0.8
84 068	09/09/84	19.90	Female	4.40	18.6	19.0	0.4	0.6	0.6
84 078	17/09/84	20.10	Male	1.07	18.8	19.6	0.8	0.8	0.8
84 088	27/09/84	19.10	Female	1.84	18.5	19.1	0.6	0.7	0.8
85 022	10/09/85	17.30	Male	1.94	18.3	18.8	0.5	0.6	0.6
85 025	18/09/85	18.30	Female	1.93	19.6	19.6	0.0	0.8	0.9

**FIGURE 1:**
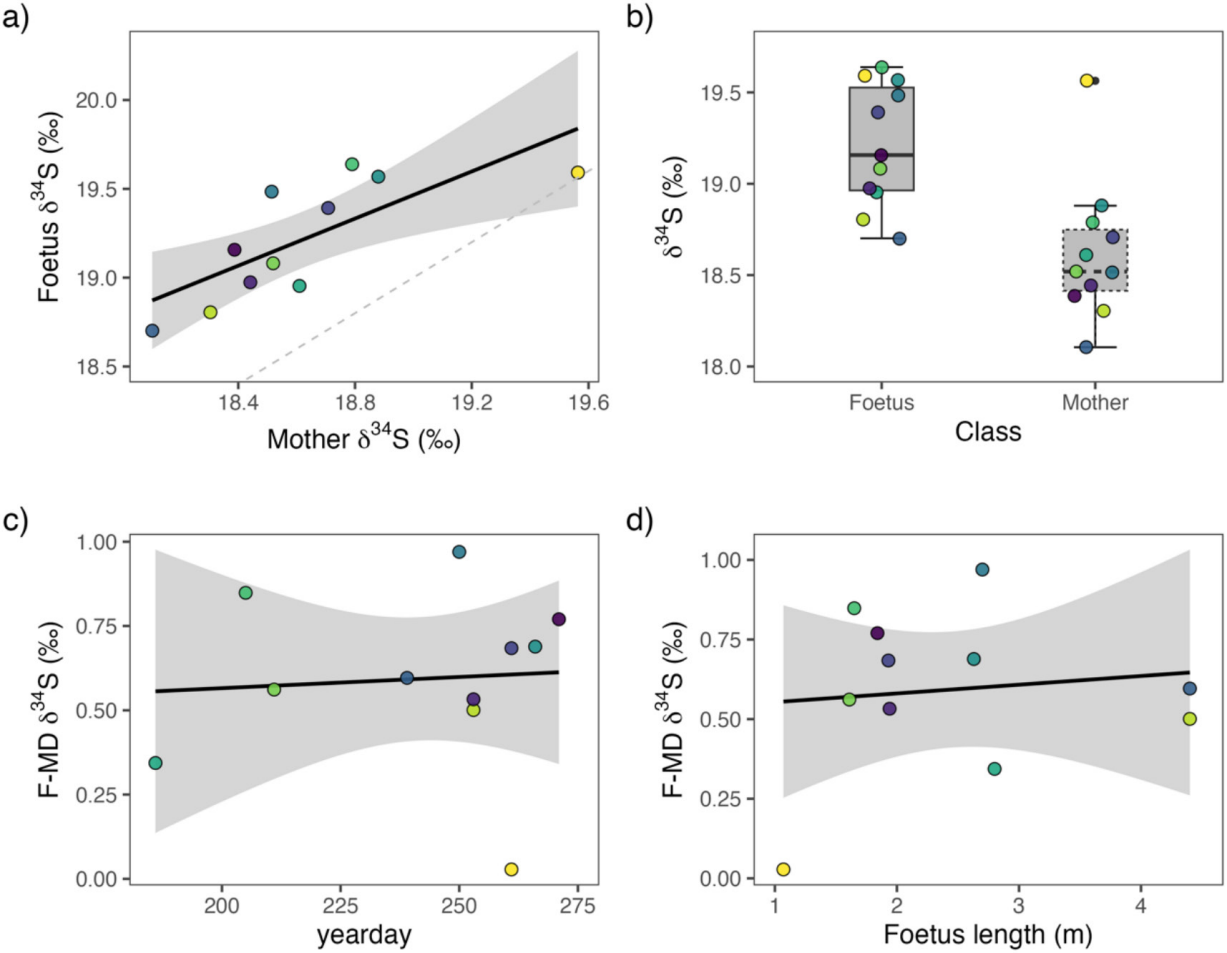
(a) Plot illustrating the linear relationship between *δ* ^34^S values of the fin whale foetuses and their mothers (*R* ^2^_adj_. = 0.52; F (1, 9) = 11.99; *p* = 0.007). (b) Boxplot depicting overall differences between muscle *δ* ^34^S values from fin whale foetuses and mothers. Note that foetuses are enriched in ^34^S relative to their mothers (Δ^34^Sfoetus–mother = 0.59 ± 0.15 ‰). (c) Linear model (LM) showing the relationship between foetus–mother discrimination (F‐MD) of δ^34^S values and the yearday (*R* ^2^_adj._ = − 0.10; F (1, 9) = 0.04; *p* = 0.831). (d) Linear model (LM) depicting the relationship between foetus–mother discrimination (F‐MD) of *δ* ^34^S values and foetus length (in metres) (*R* ^2^_adj._ = − 0.09; F (1, 9) = 0.12; *p* = 0.731). Note that the shaded region around the fitted regression line represents the 95% confidence interval. Dot colours show the different foetus–mother pairs.

The first LM of the relationship between the *δ* ^34^S values of the fin whale foetuses and their mothers was statistically significant (*R* ^2^_adj._ = 0.52; F (1, 9) = 11.99; *p* = 0.007). Mother *δ* ^34^S values had a positive and significant effect on the foetus *δ* ^34^S values (β = 0.861 ± 0.249, *p* = 0.007; Figure 1a). The F‐MD factor for *δ* ^34^S values in fin whale muscle (Δ^34^Sfoetus–mother), or, in other words, the mean difference in *δ* ^34^S values between foetuses and their mothers, was 0.59 ± 0.15‰ and ranged from 0.0 to 1.0‰ (Figure 1b). Also, the other LMs revealed that neither the yearday (*R* ^2^_adj._ = − 0.10; F (1, 9) = 0.04; *p* = 0.831) nor the foetus length (*R* ^2^_adj._ = − 0.09; F (1, 9) = 0.12; *p* = 0.731) had a statistically significant influence on the F‐MD of *δ* ^34^S values (Figure 1c,d).

## 4 Discussion

The muscle *δ* ^34^S values in pregnant fin whales from NW Spain measured in this study (M ± SD = 18.6 ± 0.4 ‰) align with those observed in individuals of the same species from SW Iceland (M ± SD = 18.8 ± 0.4 ‰) [4] and the NW Mediterranean (M ± SD = 19.3 ± 0.4 ‰) [6]. This similarity in *δ* ^34^S values between geographically distant areas supports earlier findings indicating that sulphur isotopic compositions in open‐ocean environments are broadly homogeneous as a result of the extensive mixing of oceanic water masses, which causes similar *δ* ^34^S values of dissolved sulphates at approximately + 21 ‰ [24]. Only populations that frequent coastal waters may depart from this general scenario, as could be the case of the fin whales that feed in the Gulf of St. Lawrence during summer and fall and which venture into areas with high influence of riverine inputs [25], characterised by having lower *δ* ^34^S values than seawater. However, muscle tissue is known to integrate long periods of time, a property that may mask short‐term variability in the isotopic baseline. Thus, although no specific information on cetaceans is available, sulphur half‐life turnover rate in muscle has been estimated at 58, 110 and 173.3 days in three species of fish [26, 27, 28], 25.9 + − 9.6 days in mice [29], 55.5 + − 3.1 days in sheep [30] and 219 days in cows [31]. Likely, turnover rates in a large cetacean species such as the fin whale would be in the upper fringe of these values.

The present study evidenced that muscle *δ* ^34^S values of the fin whale foetuses and their mothers show a positive correlation. In humans, this correlation has been attributed to the sourcing of foetus protein from the mother's diet and/or tissue catabolism [13]. Also, we found that *δ* ^34^S values in muscle were 0.59 ± 0.15 ‰ higher in foetuses than in their pregnant mothers.

The reason for the difference between foetal and maternal *δ* ^34^S values (F‐MD), which in this study ranged from 0.0 to 1.0 ‰, is poorly understood and likely influenced by a combination of factors. A first factor may be a different isotopic turnover rate between the muscle tissue of the foetuses and that of their mothers. It is known that tissue turnover rates can be influenced by biological traits such as age or body size [32, 33]. This may also apply to foetus/mother pairs. As North Atlantic fin whales exhibit annual oscillations in baleen *δ* ^34^S values that reflect the variation in food intake or area used [7], if the muscles of the mother and their foetuses have different turnover rates, their isotopic values may reflect different periods in habitat use and food consumption. The decoupling may also be enhanced with the growth of the foetus if the turnover rate of *δ* ^34^S values increases with foetal development. However, the finding that *δ* ^34^S values from mothers and foetuses are correlated suggests that this might not be the case or, at least, that the effect is of limited significance.

A second factor may be a different proportion of specific sulphur‐containing amino acids in the foetal and maternal muscle tissues. Variations in the catabolism of these amino acids may lead to distinct isotope fractionation if their proportions differ. As previously mentioned, cysteine can originate either directly from dietary cysteine or from dietary methionine or serine, which has been metabolised to cysteine through the transsulphuration pathway [12]. This latter process involves the transfer of the thiol group and would favour the kinetic ^34^S enrichment of foetal muscle. However, corroboration of this hypothesis requires further research.

A third and final factor may be the variation in food ingestion rates of the mother during gestation. Despite fin whales are often considered to be capital breeders, intraspecific variation in migration patterns, both in span and trajectory, is large [25] and, whatever the case, some level of feeding is known to be maintained during spring and winter [34]. This suggests that fin whales follow an intermediate strategy of energy storage involving intense feeding in summer and variable feeding or partial fasting during the rest of the year, depending on prey availability [35]. Therefore, protein in the fin whale foetus, in addition to being primarily sourced from the catabolism of maternal tissues, as would be the case in capital breeders, may also partially come from the maternal diet, as would happen in income breeders [16].

In mammals, methionine and cysteine catabolism is generally limited when food ingestion rates are low, with both sulphur amino acids being used for protein synthesis rather than for transsulphuration and oxidation reactions [36]. However, the effect of the ingestion rate on the sulphur discrimination is not well understood. On the one hand, Richards et al. [37] showed in horses that low cysteine intake could lead to the recycling of sulphur‐containing molecules and isotopic fractionation. On the other hand, Gutierrez et al. [36] showed in mice that low food intake decreased the conversion of methionine from homocysteine (re‐methylation), leading to a more direct use of methionine for protein synthesis, thus leading to lower isotope fractionation.

Fin whales may either allocate ingested nutrients or use those stored in their bodies to support foetal tissue development, leading to differential effects on the fractionation of *δ* ^34^S values during foetal tissue synthesis. In instances of reduced food intake, for example, when they are not at the high‐latitude feeding grounds, fin whales may activate transsulphuration pathways. This process would recycle sulphur‐containing molecules, potentially causing ^34^S fractionation. Additionally, given the faster rate of foetal protein synthesis, as compared to that of adults, fin whales may prioritise resource allocation towards foetal tissue growth, resulting in the observed ^34^S enrichment in the foetus relative to the mother. Similar patterns of discrimination have been observed in Southern right whales ( *Eubalaena australis* ), in which the interannual variability in offspring‐mother isotopic discrimination was attributed to the varying levels of nutritional stress each year [38].

Finally, the *δ* ^34^S F‐MD in our study were independent of yearday or foetal length and remained fairly constant throughout the development of the foetus, suggesting that the isotopic discrimination and/or routing of S‐containing amino acids across the placental barrier remains constant during the pregnancy period here studied. Further analyses using a more protracted gestation period and larger sample size would clarify this aspect and the potential impact of maternal tissue catabolism on foetal *δ* ^34^S values during winter.

These findings highlight the importance of considering F‐MD when interpreting maternal food sources and/or habitat use based on *δ* ^34^S values of foetal tissues, as well as the complexity of sulphur stable isotope dynamics, and underscore the need for further research towards a better understanding of the mechanisms driving *δ* ^34^S values fractionation in different scenarios.

## Author Contributions

**Marc Ruiz‐Sagalés:** writing – review and editing, writing – original draft, visualization, investigation, formal analysis, conceptualization, methodology, software, data curation, validation. **Asunción Borrell:** writing – review and editing, funding acquisition, project administration, resources, investigation, supervision, conceptualization, methodology, validation, data curation. **Alex Aguilar:** writing – review and editing, funding acquisition, project administration, resources, investigation, supervision, conceptualization, methodology, validation, data curation.

### Peer Review

The peer review history for this article is available at https://www.webofscience.com/api/gateway/wos/peer‐review/10.1002/rcm.10057.

## Data Availability

The data that support the findings of this study are available from the corresponding author upon reasonable request.
